# Whole-Genome Sequence of *Weissella ceti* Strain WS08, Isolated from Diseased Rainbow Trout in Brazil

**DOI:** 10.1128/genomeA.00851-14

**Published:** 2014-08-21

**Authors:** H. C. P. Figueiredo, G. Leal, F. L. Pereira, S. C. Soares, F. A. Dorella, A. F. Carvalho, U. P. Pereira, V. A. C. Azevedo

**Affiliations:** a AQUACEN, National Reference Laboratory for Aquatic Animal Diseases, Ministry of Fisheries and Aquaculture, Federal University of Minas Gerais, Belo Horizonte, Brazil; b Laboratory of Cellular and Molecular Genetics, Institute for Biological Science, Federal University of Minas Gerais, Belo Horizonte, Brazil

## Abstract

We report here the complete genome sequence of *Weissella ceti* strain WS08, an emerging pathogen to farm-raised rainbow trout. The genome of strain WS08 is composed of a circular chromosome with 1,355,853 bp and a G+C content of 40.78%.

## GENOME ANNOUNCEMENT

*Weissella ceti* is a Gram-positive bacterium that is the etiologic agent of weissellosis in farm-raised rainbow trout, *Oncorhynchus mykiss* (Walbaum, 1792) (1, 2). This emergent disease occurs mainly during summer season, and it is characterized by acute hemorrhagic septicemia and high mortality (3). Outbreaks caused by *W. ceti* have been reported from farms in China, Brazil, and the United States (1–4). In 2013, the first genome sequence of *W. ceti* isolated from rainbow trout was published (5), which was deposited as a draft genome containing eight unordered contigs.

DNA from *W. ceti* strain WS08 was isolated from overnight culture with the Maxwell 16 tissue DNA purification kit using the Maxwell 16 system (both from Promega). The genome was sequenced with the PGM Ion Torrent sequencing system (Life Technologies, Carlsbad, CA) using a combination of fragment and mate-paired library approaches. An average insert length of 6,000 bp was used in a mate-pair library. Both libraries were sequenced with the Ion PGM 200 sequencing kit, according to the manufacturer’s recommendations. A total of 1.9 million fragment reads and 5.8 million mate-paired reads were obtained, with coverages of ~230× and ~640×, respectively. The fragment reads were assembled with Mira Assembler version 3.9.18 (*N*_50_, 49,267 bp) using the recommended parameters (6). Ten contigs with an average length of 93 kbp were assembled. For the mate-pair data, Newbler Assembler version 2.9 (454 Sequencing) was applied and resulted in 60 scaffolds (*N*_50_, 31,809 bp). BLASTn (http://blast.ncbi.nlm.nih.gov/) and in-house scripts were used to evaluate the overlapped contigs. The obtained contigs were scaffolded, and the remaining gaps were resolved with CLC Genomics Workbench version 5.5.1 (CLC bio, Germantown, MD) by gap filling with recursive mappings against the scaffold.

*W. ceti* strain WS08 is composed of a circular chromosome with 1,355,853 bp and a G+C content of 40.78%. The genome was automatically annotated using Prokka version 1.7 (7). A total of 1,365 genes were annotated, representing 1,269 putative coding sequences, one pseudogene with a frameshift mutation compared with the genome sequence of *W. ceti* NC36 (5), 19 rRNAs (5S, 16S, and 23S), representing 6 complete rRNA operons plus an additional 5S gene, 75 tRNAs (2 tRNAs were undetermined), and one transfer-messenger RNA (tmRNA).

The obtained genome sequence provides a solid basis for the functional genomic analysis as well as the comparison between the *W. ceti* strains isolated from different regions.

### Nucleotide sequence accession numbers.

This whole-genome shotgun project has been deposited at GenBank under the accession no. CP007588. The version described in this paper is the first version, CP007588.1.
